# Genetic differentiation and diversity of *Callosobruchus chinensis* collections from China

**DOI:** 10.1017/S0007485315000863

**Published:** 2015-11-09

**Authors:** C.X. Duan, W.C. Li, Z.D. Zhu, D.D. Li, S.L. Sun, X.M. Wang

**Affiliations:** 1 Institute of Crop Science, Chinese Academy of Agricultural Sciences/National Key Facility for Crop Gene Resources and Genetic Improvement, Beijing 100081, China; 2 College of Life Sciences, Henan Normal University, Xinxiang 453007, China

**Keywords:** *Callosobruchus chinensis*, simple sequence repeat, geographic populations, genetic differentiation, genetic diversity

## Abstract

*Callosobruchus chinensis* (Linnaeus) is one of the most destructive pests of leguminous seeds. Genetic differentiation and diversity analysis of 345 *C. chinensis* individuals from 23 geographic populations using 20 polymorphic simple sequence repeats revealed a total of 149 alleles with an average of 7.45 alleles per locus. The average Shannon's information index was 1.015. The gene flow and genetic differentiation rate values at the 20 loci ranged from 0.201 to 1.841 and 11.0–47.2%, with averages of 0.849 and 24.4%, respectively. In the 23 geographic populations, the effective number of alleles and observed heterozygosity ranged from 1.441 to 2.218 and 0.191–0.410, respectively. Shannon's information index ranged from 0.357 to 0.949, with the highest value in Hohhot and the lowest in Rudong. In all comparisons, the fixation index (*F*_*ST*_) values ranged from 0.049 to 0.441 with a total *F*_*ST*_ value of 0.254 among the 23 *C. chinensis* populations, indicating a moderate level of genetic differentiation and gene flow among these populations. Analysis of molecular variance revealed that the genetic variation within populations accounted for 76.7% of the total genetic variation. The genetic similarity values between populations varied from 0.617 to 0.969, whereas genetic distances varied from 0.032 to 0.483. Using unweighted pair-group method using arithmetical averages cluster analysis, the 23 geographic collections were classified into four distinct genetic groups but most of them were clustered into a single group. The pattern of the three concentrated groups from polymerase chain reactions analysis showed a somewhat different result with cluster.

## Introduction

As a traditional human food resource, legumes are important crops for planting structure adjustment, international trade and increasing the income of mountain farmers in China. In recent years, human health consciousness has increased and the nutritive value of legumes has been recognized. Thus, legumes have increasingly become the focus of agricultural production. China ranks first in the world for food legume yield and number of types produced. Also, as the world's second-biggest exporter, China accounts for 12% of global food legume exports (Liu, 2012). Therefore, to a certain degree, edible beans have great competitive advantage and development potential for food production in the mountains and the international market.

*Callosobruchus chinensis* L. (Coleoptera: Bruchidae) is one of the most serious pests of leguminous stored seeds in Asia (Somta *et al*., 2008). The larvae of *C. chinensis* use a variety of dried legume seeds as hosts, such as mung bean (*Vigna radiata* L.) (Shinoda *et al*., 1991), cowpea (*Vigna unguiculata* L.) (Tomooka *et al*., 2000), adzuki bean (*Vigna angular* Ohwi & Ohashi) (Tomooka *et al*., 2000), faba bean (*Vicia faba* L.), pea (*Pisum sativum* L.), soybean (*Glycine max* L. Merr.) (Duan *et al*., 2014*a*), chickpea (*Cicer arietinum* L.), pigeon pea [*Cajanus cajan* (L.) Millspaugh] (Nahdy *et al*., 1998), kidney bean (*Phaseolus vulgaris* L.), peanut (*Arachis hypogaea* L.), and lotus seed (*Nelumbo nucifera* Gaertn.) (Li & Zhu, 2009). As is typical of the life history of the Bruchidae, these larvae bore into legume seeds upon hatching and feed on the seed and consume the cotyledons (Tuda *et al*., 2004). They pupate and metamorphose into adults within the seed and emerge to seek mates and new hosts, which causes secondary infestation, often causing considerable damage to stored food legumes (Tuda *et al*., 2004). Usually, the afflicted legumes show an infestation percentage of 30–64%, but this may be as high as 80–100%, which significantly decreases or even completely eliminates the 1000-kernel weight, nutritive value and germination rate, making them unfit for human consumption or for agricultural and commercial use (Umrao & Verma, 2002; Chaubey, 2008; Duan *et al*., 2014*a*).

Simple sequence repeats (SSRs) or microsatellite DNA markers are extensively used in the construction of genetic linkage maps, the evaluation of genetic diversity, ecology and evolution studies, and many other types of studies. Microsatellites are popular genetic markers because of their co-dominance, high abundance and polymorphism rates, multi-allelic nature, and rapid detection of alleles by a wide variety of methods (Queller *et al*., 1993; Ellegren, 2004; Choudhary *et al*., 2009; Dutta *et al*., 2011; Duan *et al*., 2014*b*). In recent years, SSR markers have been increasingly widely used in genetic variation studies of insect populations (Behura, 2006), such as genetic variation, genetic diversity and phylogenetic relationship analyses of silkworm (*Bombyx mori*) (Li *et al*., 2005*a*, *b*), Mediterranean fruit fly (*Ceratitis capitata*) (Bonizzoni *et al*., 2001), bumble bee (*Bombus hypnorum*) (Paxton *et al*., 2001), Argentine ant (*Linepithema humile*) (Tsutsui *et al*., 2003), wild silkworm (*Bombyx mandarin*) (Li *et al.*, 2005*a*), *Cacopsylla chinensis* Yang et Li (Sun *et al*., 2011), biotype Q whitefly (*Bemisia tabaci* Gennadius) (Tsagkarakou *et al*., 2012), brown planthopper (*Nilaparvata lugens* Stål) (Jing *et al*., 2012), *Cydia pomonella* L. (Gund *et al*., 2012), silkmoth (*Antheraea assamensis* Helfer) (Singh *et al*., 2012), fall armyworm (*Spodoptera frugiperda*) (Pavinato *et al*., 2013) and soybean aphid (*Aphis glycines* Matsumura) populations (Jun *et al*., 2013).

To date, research reports about the genetic diversity of *C. chinensis* have been rare. A 522-bp fragment of the mitochondrial cytochrome c oxidase I (COI) gene was sequenced for genetic diversity analysis of eight populations of *C. chinensis* from Japan, Korea and Taiwan collected from different habitats, and six haplotypes were detected based on the COI sequence differences (Tuda *et al*., 2004). By combining the sequence differences of the mitochondrial DNA (mtDNA) COI gene, cytochrome b gene (Cytb) and the second internal transcribed spacer of the nuclear ribosomal DNA (rDNA ITS2) of 12 geo-populations of *C. chinensis* and three other species, the phylogenetic relationships among different populations and species were analyzed and discussed (Xu, 2007). However, no studies of the population genetic structure and genetic diversity of *C. chinensis* using SSR markers have been reported to date.

We conducted a national survey of the distribution and degree of damage of *C. chinensis* to stored edible beans during 2011–2013 and obtained populations from diverse geographic regions. Subsequently, high-put sequencing of the *C. chinensis* genome was performed with the Illumina HiSeq2500 platform and more than 6300 pairs of SSR primers were developed, which provided a great tool for the study of *C. chinensis* population variation and genetic diversity (Duan *et al*., 2014*b*). Based on this preliminary work, 20 pairs of polymorphic SSR primers were selected to analyze the genetic diversity of 23 different *C. chinensis* geographic populations. The aim of this study was to elucidate the population structure and genetic differences of *C. chinensis* from different parts of China, which will provide valuable information for *C. chinensis*-resistance breeding.

## Materials and methods

### *C. chinensis* population

**Fig. 1.**
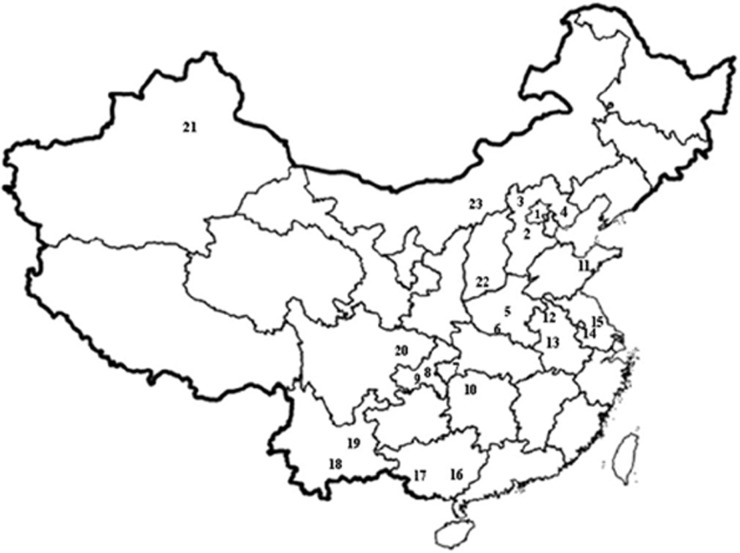
Locations of the 23 *Callosobruchus chinensis* populations in China. Numbers represent the different populations. 1: Mengtougou, Beijing (MT); 2: Baoding, Hebei (BD); 3: Yangyuan, Hebei (YY); 4: Tangshan, Hebei (TS); 5: Xinye, Henan (XY); 6: Dengzhou, Henan (DZ); 7: Enshi, Hubei (ES) ;8: Qianjiang, Chongqing (QJ); 9: Wulong, Chongqing (WL); 10: Changde, Hunan (CD); 11: Qingdao, Shandong (QD); 12: Mengcheng, Anhui (MC); 13: Hefei, Anhui (HF); 14: Lishui, Jiangsu (LS); 15: Rudong, Jiangsu (RD); 16: Bobai, Guangxi (BB); 17: Daxin, Guangxi (DX); 18: Jiangchuan, Yunnan (JC); 19: Luliang, Yunnan (LL); 20: Guangan, Sichuan (GA); 21: Urumuqi, Xinjiang (UM); 22: Lingchuan, Shanxi (LC); 23: Hohhot, Inner Mongolia (HH).

The pests were collected from mungbean or adzuki bean seeds preserved in different regions and then stored at −30°C. Information on the *C. chinensis* collections is shown in fig. 1.

### Genomic DNA extraction

The total genomic DNA of a single adult with its gut removed was extracted with the TIANamp Genomic DNA Kit according to the manufacturer's instructions (TIANGEN, Beijing, China). The quality of the extracted DNA was monitored on 1.5% agarose gels. DNA purity was checked using the NanoPhotometer^®^ spectrophotometer (Implen, San Diego, CA, USA). The DNA concentration was measured using a Qubit^®^ DNA Assay Kit in a Qubit^®^ 2.0 Fluorometer (Life Technologies, San Diego, CA, USA). The DNA was stored at −20°C for subsequent analyses.

### Simple sequence repeat primers

The SSR primers used in the present study were developed by Duan *et al*. (2014*b*). The primers were synthesized by Sangon Biotech Co., Ltd. (Shanghai, China). A total of 20 polymorphic SSR loci were selected for genetic diversity analysis. The characteristics of the primers are shown in table 1.

**Table 1.** Characteristics of 20 polymorphic SSR markers used in genetic diversity analysis (F, forward primer, R, reverse primer, Size, size of cloned allele, Ta, annealing temperature).

Primer	SSR motif	F (5′–3′)	R (5′–3′)	Region	Size (bp)	*Ta* (°C)
CCM1	AACA	TTGGTGCTAACGTACATGAAATGAA	CTCGCATAACGACTTCATGGATTT	Coding	155	58
CCM2	TAT	CAGGACTGGACTGGACATGTGATA	TGCTCAGCCTAATTCAACCTGTTT	Noncoding	156	55
CCM4	ATA	CTGAACCGAATTTGAGCCGTAATA	CATTCAGATGTTTCCATCTGCTTCT	Noncoding	133	55
CCM16	TTA	GAAATACGAGCATTCGGTCAAAAT	CCTTCGAGGTGGCTCTATTTGTAT	Noncoding	104	55
CCM29	TA	CAATGGCATGTCCACCACTACT	GATAAGCAATTGCCTCTTATCCCA	Noncoding	146	55
CCM33	ATA	ACGACTTTATCCACAGAAGCCAAT	TAATATTCATGGACTTGGAGGCGT	Coding	141	55
CCM40	TAT	AGGGGATATCGTGTTCTCTGTTCA	GATCGAACCTCGAAGCAGAAGTT	Noncoding	115	55
CCM46	AGAC	AACGGACAGACAGAGAGATAGTCAA	TCTGTTTGTTTGCCTGTTTGC	Coding	134	55
CCM49	TATC	GTTGACCGTTGGACAATAAAGCTA	TGTCACTACAATCGATTCGTCTCAT	Coding	145	55
CCM56	TG	CCTAAGAGTTTCGTTTTTGCATGG	GGCAAATTGAGCTCAGCATTAGAT	Coding	110	55
CCM71	CGA	GCACTATACTCAGGACGTCTCGC	CGACGTCTCATCTCTTCCAGGTAT	Noncoding	132	55
CCM72	TAA	TGCATATGTCCAAGAACAATCTCG	ACTCTACATCCCCTGGAGCGTTAT	Coding	133	55
CCM83	AAT	AGGGTTTTTCCCGTTTAACGATATT	CGGATCTGGAAGCAGTTTTGTTTA	Coding	140	57
CCM108	TGTA	ACTTAAATTACCCGGTGACGTGAA	AACTTTTGTAAATCGCGCTCAAAG	Coding	131	55
CCM118	TGT	TTAATTCTGGTCCTGTTCCGTTTT	TCTTCTAATCCCATGACTAGCTTCAC	Noncoding	130	55
CCM125	GAA	AGCGTGTTAAAAAGAGAGTGGACG	TATTATTCTGCAGATGTGCCGCT	Coding	144	57
CCM139	TAT	ACTGCTGCCTTAAAAAGAAAGGCT	AAGGAACGGTTAGCACTTGGATG	Coding	141	55
CCM143	TAA	TTCGAAGAATTCTAAGCGTATGTGC	CGGTTGAGTAGAAGTAGCCGTTGT	Noncoding	133	55
CCM162	TACA	GTAAATCGCGCTCAAAGATTCAGT	TCAACACAAACATGCATCAAAAGA	Coding	113	56
CCM168	ATA	ATAGGTTGTGGGAGTCTGAAGGTG	AGTGGTCAAGAAGGACTATGTCGG	Coding	138	55

### Polymerase chain reaction (PCR) amplification and product detection

Simple sequence repeat analysis was performed following the procedure of Duan *et al*. (2014*b*). PCR were carried out in 10 µl reaction volumes containing 10 mmol l^−1^ Tris-HCl pH 8.3, 50 mmol l^−1^ KCl, 1.5 mM MgCl_2_, 250 mM of each dNTP, 0.2 M of each primer, 0.5 U Taq polymerase (Dingguo, Beijing, China) and 30 ng of DNA template. Reactions were performed using a GeneAmp^®^ PCR System 9700 thermal cycler (ABI, Norwalk, CT, USA) programmed for 94°C for 4 min, followed by 35 cycles of 94°C for 30 s, 50–60°C (depending on the optimized annealing temperature) for 30 s and 72°C for 60 s. A final extension was performed at 72°C for 10 min. The PCR products were analyzed by electrophoresis on 8.0% non-denaturing polyacrylamide gels and silver stained. The band sizes were determined roughly based on a 100 bp DNA ladder (TIANGEN, Beijing, China).

### Data analysis

The fragment lengths of the alleles from each individual were recorded from the banding patterns of SSR amplifications. In order to reduce genotyping errors of microsatellites, MICROCHECKER 2.2.3 soft was used to remove or adjust null alleles (van Oosterhout *et al*., 2004). Data analysis was performed using the population genetics analysis software PopGene32 (version 1.31). This included determination of the number of alleles (*Na*), number of effective alleles (*Ne*), observed heterozygosity (*Ho*), expected heterozygosity (*He*), Nei's expected heterozygosity (*Nei*), Shannon's information index (*I*), F-statistics (*F*_*IS*_, *F_IT_, F_ST_*), gene flow (*N*_*m*_), Nei's genetic identity and genetic distance (*D*) between various geographical populations. Significance analysis was performed with SPSS10.0 software (SPSS Inc., Chicago, IL).

Population differentiation was tested by comparing allele frequencies among 23 populations using Weir and Cockerham's *F*_*ST*_ (θ) value (Weir & Cockerham, 1984). The *F*_*ST*_-value was estimated under the null hypothesis of non-differentiation among subpopulations, where *F*_*ST*_ = 0. Statistical analysis was done by comparing the calculated *F*_*ST*_ values with those of datasets in which individual bean weevils were randomized across populations 10,000 times using the programme Multilocus 1.3b (Agapow & Burt, 2001). Gene flow (*Nm*) was determined using the equation *Nm* = 0.5(1 − *F*_*ST*_)/*F*_*ST*_ (McDermott & McDonald, 1993; Asadollahi *et al*., 2013). Analysis of molecular variance (AMOVA) was used to partition the total genetic variance within and among populations from different geographic regions (Isenegger *et al*., 2008). The AMOVA was implemented in the GENALEX 6 program (Peakall & Smouse, 2006).

The bootstrap analysis was performed with 1000 replicates for statistical support of branches in unweighted pair-group method using arithmetical averages (UPGMA) clustering trees using the MEGA 4 software (Tamura *et al*., 2007). Principal component analysis (PCA) and three-dimensional (3D)-PCA graph were performed based on Euclid distance among 345 *C. chinensis* collections using NTSYS-pc software package V2.2 (Rohlf, 1998).

## Results

### Genetic polymorphism of the microsatellite loci

**Fig. 2.**
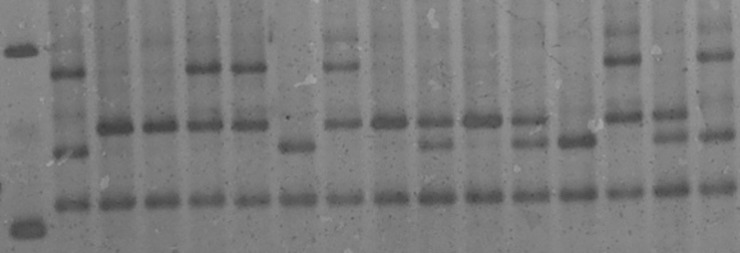
PCR amplification patterns of 15 *Callosobruchus chinensis* individuals from QD population using primer CCM46 (the left lane 1 is the 100 bp DNA ladder).

MICRO-CHECKER estimated 0.069 of frequency of null alleles, which coincided with the excellent amplification of these microsatellite loci among *C. chinensis*. In all, 345 *C. chinensis* individuals were genotyped at 20 microsatellite loci, which were used to evaluate the genetic diversity and differentiation among 23 *C. chinensis* populations (table 2 and fig. 2). A total of 149 alleles were detected among the 20 SSRs, ranging from 4 to 13 alleles per locus with an average of 7.45 (table 2). The effective number of alleles per locus varied from 1.521 to 4.845, with an average of 2.640. The expected and observed heterozygosities ranged from 0.166 to 0.799 and 0.130 to 0.658, with averages of 0.503 and 0.401, respectively. The values of Nei's expected heterozygosity and Shannon's information index varied from 0.155 to 0.793 and 0.353 to 1.767, with averages of 0.500 and 1.015, respectively (table 2). In addition, the observed heterozygosity significantly deviated from expected heterozygosity at loci CCM46 and CCM118 (*P* < 0.05).

**Table 2.** The genetic variation among twenty microsatellite loci of *Callosobruchus chinensis* in China.

Locus	Sample size	*Na*	*Ne*	Ho*	He*	*Nei*	*I*
CCM1	666	9	3.605	0.598 a	0.755 a	0.753	1.553
CCM2	690	6	2.140	0.130 a	0.166 a	0.155	0.353
CCM4	680	5	1.924	0.297 a	0.356 a	0.327	0.649
CCM16	662	8	2.435	0.417 a	0.590 a	0.589	1.178
CCM29	676	13	3.380	0.388 a	0.496 a	0.495	1.092
CCM33	672	4	1.521	0.384 a	0.343 a	0.342	0.682
CCM40	666	6	2.465	0.360 a	0.434 a	0.431	0.908
CCM46	656	7	3.298	0.497 a	0.698 b	0.697	1.423
CCM49	686	7	2.310	0.536 a	0.568 a	0.567	1.025
CCM56	646	13	3.268	0.658 a	0.695 a	0.693	1.503
CCM71	666	4	2.022	0.351 a	0.506 b	0.505	0.776
CCM72	650	7	1.786	0.195 a	0.285 a	0.282	0.677
CCM83	672	9	2.926	0.339 a	0.345 a	0.348	0.748
CCM108	646	8	2.196	0.395 a	0.546 a	0.544	0.951
CCM118	658	9	4.845	0.550 a	0.799 b	0.793	1.767
CCM125	686	7	2.332	0.302 a	0.416 a	0.415	0.798
CCM139	646	5	1.881	0.134 a	0.178 a	0.172	0.462
CCM143	632	6	2.590	0.495 a	0.611 a	0.613	1.188
CCM162	666	9	3.680	0.598 a	0.729 a	0.723	1.472
CCM168	684	7	2.191	0.395 a	0.554 a	0.551	1.091
Mean	665	7.45	2.640	0.401 a	0.503 a	0.500	1.015

*Na* and *Ne* are the average number of alleles and effective number of alleles per locus, respectively. *Ho* and *He* are the observed and expected heterozygosity, respectively. *Nei* is Nei's expected heterozygosity. *I* is Shannon's information index.

* Significance analysis was conducted between *Ho* and *He* values. The same lowercase (a) in the same row showed no significant difference between *Ho* and *He* values at the locus.

The genetic differentiation among populations was assessed using fixation indices (*F*_*IS*_, *F_IT_, F_ST_*) and measuring gene flow (*N*_*m*_) at each locus. The F-statistics at each locus among the 23 populations are listed in table 3. The gene flow and genetic differentiation rate values at the 20 loci ranged from 0.201 to 1.841 and 11.0–47.2%, with averages of 0.849 and 24.4%, respectively. It was found that 12 loci, including CCM72, CCM118, CCM1, CCM83, CCM29, CCM56, CCM125, CCM143, CCM71, CCM46, CCM108 and CCM16, had high genetic differentiation among populations (*N*_*m*_ < 1). The other eight loci, including CCM139, CCM168, CCM2, CCM40, CCM49, CCM33, CCM162 and CCM4, showed 1 < *N*_*m*_ < 2; none of the loci had low genetic differentiation among populations (*N*_*m*_ > 4). This indicated that the loci CCM72, CCM118, CCM1, CCM83, CCM29, CCM56, CCM125, CCM143, CCM71, CCM46, CCM108 and CCM16 had relatively large genetic differentiation among populations while CCM139, CCM168, CCM2, CCM40, CCM49, CCM33, CCM162 and CCM4 had moderate genetic differentiation (table 3).

**Table 3.** The results of *F*-statistics analysis and gene flow (*Nm*) among the loci.

Locus	*F*_*IS*_	*F*_*IT*_	*F*_*ST*_	*Nm*
CCM1	0.493	0.645	0.307	0.396
CCM2	−0.071	0.100	0.160	1.307
CCM4	−0.199	0.004	0.170	1.225
CCM16	0.411	0.554	0.201	0.765
CCM29	0.161	0.422	0.308	0.503
CCM33	−0.366	−0.120	0.180	1.139
CCM40	−0.012	0.187	0.196	1.024
CCM46	0.130	0.340	0.242	0.780
CCM49	−0.163	0.055	0.187	1.086
CCM56	−0.326	0.060	0.291	0.610
CCM71	0.067	0.305	0.255	0.730
CCM72	0.212	0.573	0.472	0.201
CCM83	−0.456	0.0249	0.330	0.507
CCM108	0.235	0.413	0.228	0.807
CCM118	0.180	0.565	0.465	0.278
CCM125	0.791	0.821	0.203	0.534
CCM139	0.455	0.512	0.110	1.841
CCM143	0.241	0.453	0.278	0.623
CCM162	0.195	0.333	0.168	1.165
CCM168	0.398	0.484	0.138	1.456
Mean	0.119	0.337	0.244	0.849

### Genetic differentiation and diversity among *C. chinensis* populations

The genetic diversity of *C. chinensis* from the 23 geographic populations is shown in table 4. In the 23 geographic populations, the number of alleles and effective number of alleles per population ranged from 1.800 to 3.200 and 1.441 to 2.218, respectively, with the observed heterozygosity varying from 0.191 to 0.410, the expected heterozygosity ranging from 0.230 to 0.431 and Nei's expected heterozygosity ranging from 0.223 to 0.417. Shannon's information index ranged from 0.357 to 0.949, with the highest value in Hohhot (HH), Inner Mongolia and the lowest in Rudong (RD), Jiangsu.

**Table 4.** The genetic variation statistics among 23 *Callosobruchus chinensis* populations.

Population	*Na*	*Ne*	*Ho*	*He*	*Nei*	*I*
XY	3.050 ± 2.038	2.218 ± 1.655	0.349 ± 0.310	0.406 ± 0.289	0.391 ± 0.278	0.663 ± 0.406
HH	2.700 ± 1.218	1.933 ± 0.883	0.330 ± 0.275	0.409 ± 0.231	0.395 ± 0.142	0.949 ± 0.393
RD	1.850 ± 1.040	1.441 ± 0.529	0.238 ± 0.295	0.230 ± 0.253	0.223 ± 0.245	0.357 ± 0.396
YY	2.700 ± 1.380	1.999 ± 0.889	0.325 ± 0.280	0.414 ± 0.264	0.399 ± 0.255	0.680 ± 0.464
QD	2.850 ± 1.598	1.900 ± 0.875	0.293 ± 0.268	0.381 ± 0.266	0.368 ± 0.257	0.653 ± 0.487
MC	2.550 ± 1.099	1.772 ± 0.669	0.307 ± 0.291	0.377 ± 0.218	0.365 ± 0.210	0.610 ± 0.368
UM	2.400 ± 0.995	1.789 ± 0.587	0.306 ± 0.309	0.386 ± 0.238	0.373 ± 0.230	0.599 ± 0.375
QJ	2.550 ± 0.759	1.648 ± 0.418	0.350 ± 0.263	0.365 ± 0.184	0.351 ± 0.177	0.577 ± 0.270
LL	2.550 ± 1.050	1.776 ± 0.534	0.338 ± 0.221	0.399 ± 0.199	0.385 ± 0.192	0.641 ± 0.338
LC	2.650 ± 1.226	1.848 ± 0.721	0.311 ± 0.286	0.390 ± 0.245	0.376 ± 0.237	0.636 ± 0.417
LS	2.600 ± 0.883	1.826 ± 0.573	0.342 ± 0.240	0.414 ± 0.199	0.399 ± 0.191	0.652 ± 0.326
HF	2.050 ± 1.191	1.475 ± 0.553	0.257 ± 0.331	0.247 ± 0.251	0.238 ± 0.242	0.390 ± 0.399
BD	2.600 ± 1.392	1.806 ± 0.838	0.221 ± 0.265	0.348 ± 0.275	0.336 ± 0.266	0.595 ± 0.491
DZ	2.650 ± 0.813	1.864 ± 0.597	0.378 ± 0.234	0.430 ± 0.186	0.414 ± 0.179	0.686 ± 0.308
DX	2.600 ± 1.0463	1.935 ± 0.760	0.277 ± 0.246	0.412 ± 0.249	0.398 ± 0.241	0.677 ± 0.416
JC	3.200 ± 1.399	1.922 ± 0.696	0.265 ± 0.272	0.417 ± 0.244	0.402 ± 0.235	0.721 ± 0.434
BB	2.450 ± 0.945	1.822 ± 0.635	0.284 ± 0.285	0.397 ± 0.229	0.382 ± 0.221	0.623 ± 0.367
TS	2.100 ± 0.788	1.561 ± 0.533	0.191 ± 0.235	0.301 ± 0.228	0.291 ± 0.220	0.470 ± 0.351
MT	2.100 ± 0.718	1.696 ± 0.452	0.266 ± 0.294	0.373 ± 0.209	0.361 ± 0.202	0.558 ± 0.321
ES	2.800 ± 1.765	2.100 ± 1.080	0.410 ± 0.330	0.431 ± 0.257	0.417 ± 0.249	0.713 ± 0.484
WL	2.250 ± 0.550	1.679 ± 0.332	0.381 ± 0.298	0.390 ± 0.158	0.376 ± 0.152	0.577 ± 0.215
CD	1.800 ± 0.768	1.500 ± 0.418	0.297 ± 0.350	0.286 ± 0.225	0.276 ± 0.217	0.416 ± 0.328
GA	2.350 ± 0.933	1.588 ± 0.432	0.290 ± 0.279	0.336 ± 0.187	0.325 ± 0.181	0.532 ± 0.297

Genetic differentiation was analyzed by relating the genotypic distribution with *F*_*ST*_ estimates. The total *F*_*ST*_ value was 0.254 in the 23 *C. chinensis* populations, indicating a moderate level of genetic differentiation and gene flow among these populations. Pairwise comparisons of the fixation index (*F*_*ST*_) and gene flow (*Nm*) were estimated from the 20 SSR loci between *C. chinensis* populations (table 5). The highest *F*_*ST*_ value was 0.441 between the Tangshan (TS) and RD populations, followed by *F*_*ST*_ values of 0.440 and 0.430 between the Changde (CD) and TS, and CD and Guangan (GA) populations, respectively. Although the *F*_*ST*_ value between the TS and RD is greater than that between CD and TS and between CD and GA, there are no significant differences between them. The corresponding minimum *Nm* values were 0.633, 0.640 and 0.663, respectively, which suggested considerable genetic differentiation between these pairwise populations. In contrast, the *F*_*ST*_ values between the Urumuqi (UM) and Lingchuan (LC), and Enshi (ES) and Xinye (XY) populations were 0.049 and 0.050, with corresponding *Nm* values of 9.788 and 9.581, respectively, demonstrating very low genetic differentiation and sufficient gene flow between the two pairwise populations. AMOVA analysis indicated that 76.7% of the total molecular variance was due to differences within geographic regions and 23.3% of the variance was associated with differences among populations (table 6).

**Table 5.** Pairwise comparisons of fixation index (*F*_*ST*_) and gene flow (*Nm*) estimated from 20 SSR loci between populations of *Callosobruchus chinensis* from 23 geographical regions.

Population	XY	HH	RD	YY	QD	MC	UM	QJ	LL	LC	LS	HF	BD	DZ	DX	JC	BB	TS	MT	ES	WL	CD	GA
XY	–	0.189	0.380	0.228	0.213	0.276	0.205	0.235	0.282	0.212	0.198	0.346	0.331	0.182	0.225	0.275	0.256	0.336	0.321	0.050	0.298	0.357	0.334
HH	2.140	–	0.347	0.205	0.186	0.238	0.242	0.296	0.286	0.214	0.217	0.334	0.306	0.199	0.232	0.252	0.235	0.297	0.288	0.219	0.288	0.345	0.315
RD	0.815	0.941	–	0.267	0.248	0.269	0.297	0.369	0.333	0.268	0.294	0.077	0.290	0.227	0.305	0.308	0.412	0.441	0.341	0.372	0.401	0.215	0.414
YY	1.690	1.936	1.371	–	0.089	0.180	0.122	0.196	0.229	0.094	0.107	0.292	0.205	0.114	0.147	0.177	0.227	0.266	0.259	0.239	0.241	0.308	0.269
QD	1.850	2.184	1.516	5.112	–	0.073	0.139	0.197	0.234	0.094	0.093	0.238	0.194	0.102	0.116	0.151	0.233	0.251	0.229	0.226	0.262	0.274	0.262
MC	1.312	1.601	1.359	2.284	6.368	–	0.146	0.221	0.212	0.151	0.130	0.226	0.155	0.143	0.170	0.186	0.308	0.333	0.276	0.263	0.274	0.273	0.277
UM	1.938	1.568	1.185	3.585	3.107	2.934	–	0.060	0.171	0.049	0.091	0.262	0.205	0.103	0.164	0.220	0.271	0.322	0.279	0.200	0.167	0.284	0.282
QJ	1.626	1.192	0.855	2.054	2.039	1.766	7.861	–	0.220	0.123	0.103	0.339	0.272	0.110	0.204	0.286	0.321	0.336	0.319	0.200	0.101	0.345	0.348
LL	1.275	1.249	1.003	1.687	1.637	1.856	2.427	1.776	–	0.188	0.153	0.304	0.201	0.147	0.248	0.215	0.319	0.391	0.302	0.266	0.271	0.343	0.293
LC	1.860	1.832	1.368	4.797	4.802	2.816	9.788	3.555	2.160	–	0.061	0.244	0.244	0.098	0.134	0.182	0.250	0.258	0.218	0.234	0.188	0.273	0.284
LS	2.028	1.802	1.202	4.190	4.888	3.346	5.007	4.364	2.772	7.737	–	0.267	0.179	0.058	0.153	0.167	0.242	0.280	0.252	0.212	0.193	0.299	0.252
HF	0.945	0.999	6.019	1.212	1.604	1.716	1.412	0.973	1.144	1.548	1.373	–	0.287	0.226	0.316	0.297	0.395	0.426	0.329	0.343	0.392	0.114	0.394
BD	1.012	1.132	1.227	1.939	2.079	2.718	1.943	1.337	1.985	1.548	2.298	1.245	–	0.123	0.236	0.226	0.334	0.407	0.318	0.298	0.313	0.339	0.316
DZ	2.252	2.018	1.708	3.878	4.402	3.009	4.350	4.050	2.908	4.618	8.136	1.717	3.565	–	0.130	0.194	0.248	0.288	0.235	0.178	0.157	0.255	0.294
DX	1.725	1.660	1.140	2.899	3.822	2.439	2.558	1.952	1.518	3.223	2.779	1.082	1.623	3.334	–	0.126	0.183	0.302	0.262	0.221	0.232	0.332	0.221
JC	1.317	1.487	1.126	2.323	2.803	2.187	1.775	1.248	1.825	2.255	2.496	1.186	1.717	2.076	3.471	–	0.165	0.271	0.218	0.259	0.336	0.337	0.081
BB	1.452	1.627	0.713	1.700	1.647	1.124	1.344	1.058	1.067	1.498	1.568	0.767	0.997	1.517	2.240	2.530	–	0.302	0.299	0.257	0.348	0.382	0.223
TS	0.988	1.184	0.633	1.378	1.496	1.000	1.053	0.988	0.780	1.440	1.284	0.673	0.728	1.234	1.158	1.346	1.158	–	0.092	0.347	0.385	0.440	0.417
MT	1.057	1.238	0.965	1.428	1.683	1.311	1.290	1.065	1.153	1.789	1.487	1.018	1.072	1.630	1.410	1.796	1.174	4.923	–	0.327	0.355	0.366	0.377
ES	9.500	1.784	0.845	1.595	1.710	1.401	1.998	1.998	1.382	1.640	1.862	0.957	1.176	2.312	1.759	1.428	1.448	0.941	1.031	–	0.267	0.344	0.308
WL	1.179	1.237	0.748	1.571	1.407	1.322	2.490	4.455	1.346	2.157	2.088	0.777	1.096	2.689	1.653	0.987	0.935	0.799	0.909	1.373	–	0.377	0.399
CD	0.901	0.948	1.826	1.123	1.324	1.329	1.261	0.951	0.956	1.329	1.173	3.875	0.974	1.465	1.007	0.983	0.808	0.637	0.868	0.960	0.827	–	0.430
GA	0.998	1.088	0.707	1.359	1.411	1.303	1.271	0.935	1.205	1.259	1.483	0.770	1.082	1.200	1.761	5.711	1.742	0.699	0.826	1.126	0.754	0.663	–

Note: Fixation index *F*_*ST*_ (above diagonal) and gene flow *Nm* (below diagonal). *P* value < 0.001

**Table 6.** Analysis of molecular variance for 23 *Callosobruchus chinensis* populations collected from different regions in China using SSR markers.

Source of variation	Degrees of freedom	Sum of squares	Mean sum of squares	Variance components	Percentage of variation (%)	Statistics	Value	*P*-value
Among populations	22	95.643	5.508	0.438	23.3			
Within populations	323	315.800	1.592	1.592	76.7	GST	0.220	0.001
Total	345	411.443	7.100	2.03	100			

Based on the amplified fragment sizes using the 20 pairs of SSR primers, the genetic similarity and genetic distance between the 23 populations were determined (table 7). The data showed that the genetic similarity between populations varied from 0.617 to 0.969. The highest genetic similarity was found between the Hefei (HF, Anhui) and RD (Jiangsu) populations, followed by ES (Hubei) and XY (Henan), and LC (Shanxi) and Lishui (LS, Jiangsu), with genetic identity values of 0.969, 0.950 and 0.945, respectively. The lowest genetic similarity was found between the GA (Sichuan) and Wulong (WL, Chongqing) populations, followed by WL and Bobai (BB), and Luliang (LL) and TS, with genetic identity of 0.617, 0.648 and 0.651, respectively. These results suggested that the *C. chinensis* populations from HF, Anhui and RD, Jiangsu were closely related whereas those from GA, Sichuan and WL, Chongqing were relatively genetically distinct.

**Table 7.** Genetic distance and genetic identity among 23 *Callosobruchus chinensis* populations

Population	XY	HH	RD	YY	QD	MC	UM	QJ	LL	LC	LS	HF	BD	DZ	DX	JC	BB	TS	MT	ES	WL	CD	GA
XY	–	0.834	0.737	0.786	0.819	0.756	0.825	0.801	0.737	0.815	0.819	0.758	0.710	0.834	0.795	0.734	0.764	0.719	0.697	0.950	0.719	0.722	0.710
HH	0.182	–	0.753	0.811	0.843	0.790	0.781	0.731	0.719	0.810	0.797	0.753	0.723	0.810	0.780	0.754	0.783	0.765	0.733	0.787	0.725	0.717	0.722
RD	0.305	0.284	–	0.829	0.854	0.838	0.813	0.754	0.772	0.836	0.807	0.969	0.833	0.863	0.794	0.790	0.682	0.712	0.775	0.710	0.702	0.901	0.718
YY	0.241	0.210	0.187	–	0.925	0.847	0.896	0.836	0.786	0.919	0.904	0.794	0.832	0.893	0.867	0.836	0.790	0.794	0.765	0.761	0.778	0.758	0.773
QD	0.200	0.171	0.158	0.078	–	0.942	0.891	0.846	0.795	0.924	0.922	0.852	0.854	0.912	0.904	0.871	0.798	0.821	0.812	0.792	0.770	0.807	0.795
MC	0.280	0.236	0.177	0.166	0.059	–	0.885	0.825	0.820	0.880	0.893	0.863	0.887	0.879	0.857	0.841	0.712	0.737	0.763	0.750	0.757	0.808	0.780
UM	0.192	0.247	0.207	0.110	0.116	0.122	–	0.952	0.856	0.957	0.923	0.832	0.843	0.911	0.860	0.802	0.752	0.747	0.755	0.818	0.863	0.795	0.770
QJ	0.222	0.314	0.282	0.179	0.168	0.193	0.050	–	0.819	0.905	0.917	0.771	0.786	0.910	0.828	0.737	0.704	0.743	0.718	0.826	0.923	0.743	0.707
LL	0.305	0.330	0.259	0.241	0.229	0.199	0.155	0.199	–	0.838	0.866	0.787	0.842	0.868	0.766	0.801	0.684	0.651	0.719	0.734	0.751	0.724	0.754
LC	0.204	0.210	0.179	0.084	0.079	0.128	0.044	0.100	0.177	–	0.945	0.845	0.802	0.913	0.886	0.840	0.776	0.811	0.819	0.778	0.843	0.804	0.767
LS	0.200	0.227	0.214	0.101	0.081	0.113	0.080	0.086	0.144	0.057	–	0.820	0.858	0.943	0.862	0.847	0.774	0.780	0.774	0.795	0.831	0.769	0.793
HF	0.277	0.284	0.032	0.230	0.160	0.147	0.183	0.260	0.239	0.169	0.199	–	0.827	0.853	0.769	0.787	0.688	0.711	0.773	0.729	0.6981	0.948	0.726
BD	0.343	0.325	0.183	0.185	0.158	0.120	0.171	0.241	0.172	0.220	0.153	0.190	–	0.903	0.802	0.814	0.694	0.666	0.730	0.722	0.726	0.757	0.751
DZ	0.182	0.210	0.148	0.113	0.092	0.130	0.093	0.094	0.142	0.091	0.059	0.160	0.102	–	0.878	0.812	0.758	0.764	0.787	0.826	0.863	0.809	0.737
DX	0.229	0.248	0.231	0.142	0.101	0.154	0.151	0.188	0.267	0.121	0.149	0.263	0.221	0.130	–	0.888	0.839	0.756	0.763	0.783	0.789	0.730	0.823
JC	0.310	0.283	0.236	0.180	0.138	0.174	0.221	0.305	0.222	0.175	0.166	0.239	0.206	0.208	0.119	–	0.853	0.789	0.810	0.732	0.653	0.724	0.939
BB	0.270	0.244	0.383	0.240	0.226	0.340	0.286	0.352	0.381	0.254	0.256	0.374	0.365	0.277	0.176	0.159	–	0.764	0.727	0.746	0.648	0.678	0.827
TS	0.330	0.268	0.340	0.231	0.198	0.305	0.292	0.297	0.429	0.210	0.249	0.341	0.407	0.270	0.280	0.237	0.269	–	0.941	0.689	0.666	0.669	0.659
MT	0.361	0.311	0.255	0.268	0.208	0.271	0.281	0.331	0.330	0.200	0.256	0.258	0.314	0.240	0.271	0.210	0.319	0.060	–	0.667	0.654	0.712	0.661
ES	0.051	0.239	0.343	0.274	0.233	0.288	0.201	0.191	0.309	0.251	0.230	0.317	0.326	0.191	0.245	0.313	0.293	0.372	0.406	–	0.739	0.706	0.718
WL	0.330	0.321	0.353	0.251	0.261	0.278	0.147	0.081	0.287	0.171	0.186	0.359	0.321	0.148	0.237	0.427	0.433	0.406	0.424	0.303	–	0.688	0.617
CD	0.326	0.333	0.104	0.278	0.215	0.213	0.229	0.297	0.323	0.219	0.262	0.054	0.278	0.212	0.314	0.323	0.389	0.402	0.340	0.348	0.374	–	0.655
GA	0.342	0.326	0.331	0.258	0.229	0.248	0.261	0.347	0.283	0.266	0.232	0.320	0.286	0.305	0.195	0.063	0.190	0.417	0.414	0.332	0.483	0.424	–

Note: Nei's genetic identity (above diagonal) and genetic distance (below diagonal).

### Cluster analysis among *C. chinensis* populations

**Fig. 3.**
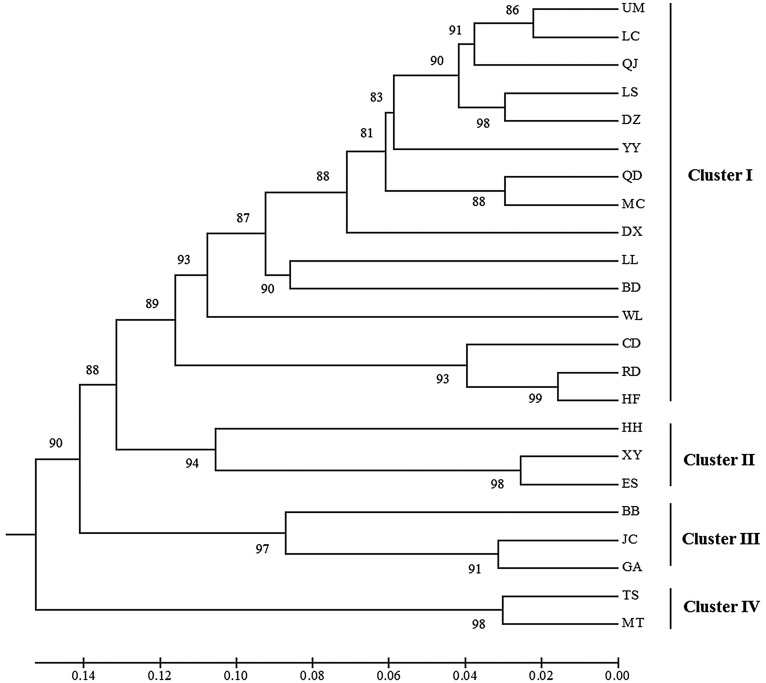
UPGMA dendrogram for 23 *Callosobruchus chinensis* populations in China based on Nei's genetic distance (numbers at branches indicate the percentage of occurrence of the cluster in 1000 bootstrapped dendrogram.).

Based on Nei's genetic distance, a UPGMA dendrogram was generated from the cluster analysis to show the genetic relationships among the 23 *C. chinensis* populations. As shown in fig. 3, the 23 populations were classified into four distinct genetic groups with a 0.12 genetic similarity coefficient. Cluster I contained 15 geographic populations, including HF, RD, CD, UM, LC, Qianjiang (QJ), LS, Dengzhou (DZ), Yangyuan (YY), Qingdao (QD), Mengcheng (MC), Daxin (DX), LL, Baoding (BD) and WL. The other eight populations were grouped into cluster II (ES, XY and HH), cluster III (GA, Jiangchuan (JC) and BB), and cluster IV (TS and Mengtougou (MT). Most of the *C. chinensis* populations were clustered into a single group, which suggested that relatively high genetic similarity existed among these populations, while there may be genetic differences among different geographic populations.

### PCA of *C. chinensis*

**Fig. 4.**
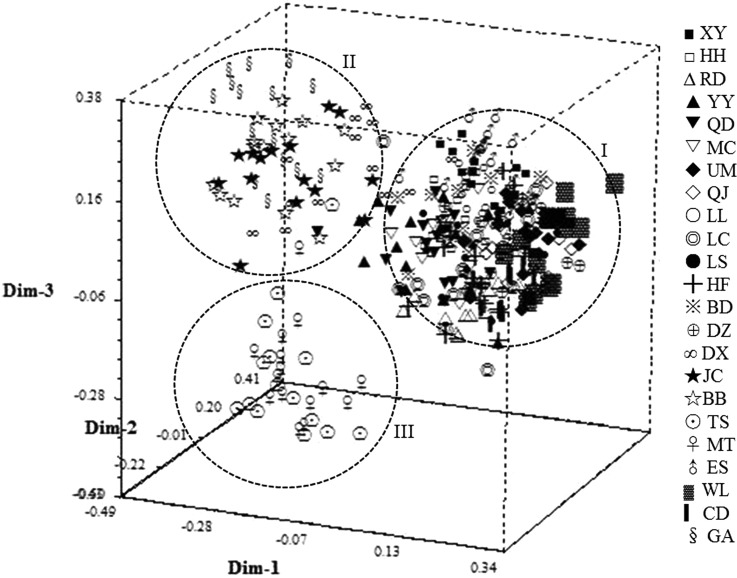
PCA and 3D-PCA graph of 345 *Callosobruchus chinensis* based on SSR analysis.

PCA of 345 *C. chinensis* collections and 3D-PCA graph were performed based on Euclid distance. The first principal components accounted for 72.7% of total variation, followed by 16.3 and 8.4%. These collections were mainly divided into three concentrated groups by geographical locations as specific component, which slightly interpenetrated with one another. The first principal component mainly contained the collections from 18 geographic populations, including HF, RD, CD, UM, LC, QJ, LS, DZ, YY, QD, MC, DX, LL, BD, WL, HH, XY and ES. The second principal component was mainly composed of *C. chinensis* from GA, JC and BB populations. The third merely contained collections from TS and MT (fig. 4). The pattern of the three major groups from PCA showed a somewhat different result with cluster. The first principal component corresponds to the Cluster I and Cluster II. However, the second and third components coincide with the Cluster III and Cluster IV, respectively.

## Discussion

*C. chinensis* is a destructive storage pest of legume beans that often causes considerable loss in the quantity and quality of seeds during transportation and storage. This pest widely is distributed around China and has multiple hosts, showing strong environmental adaptability. However, a lack of genetic information about this pest has meant that a series of genetic questions remain largely unanswered, such as its population genetic structure, genetic differentiation, kinship and biotype abundance. An understanding of population genetic structure and differentiation is important when devising pest or disease management and resistance-screening strategies. For example, a study of the chickpea pathogen *Ascochyta rabiei* found that significant genetic differentiation existed between disease nursery populations and populations in commercial fields, demonstrating that screening for resistance in the disease nursery may not have exposed plants to appropriate isolates of the pathogen (Peever *et al*., 2004). Moreover, some different resistance genes should be considered for resistance breeding in order to control effectively pest or pathogen populations with high genetic variation. On the contrary, the variety with a certain target resistance gene could be deployed and cultivated in different regions suffering from a certain pest. Therefore, the clarification of the genetic diversity of pest or pathogen populations makes sense in enacting resistance breeding strategy. The genetic diversity of several populations of *C. chinensis* from Japan, Korea and Taiwan was analyzed via polymorphism in mitochondrial COI gene sequences (Tuda *et al*., 2004; Xu, 2007), but the number of *C. chinensis* populations examined and the genetic variation information provided by COI sequence differences was limited.

Microsatellite loci are powerful tools for population genetics studies because of the reproducibility and precision of these markers in detecting multiple alleles. Here, we used 20 microsatellite markers developed by Duan *et al*. (2014*b*) using illumina paired-end sequencing of *C. chinensis* genome to determine the genetic diversity and structure of 23 *C. chinensis* geographic populations from China. Actually, we firstly selected more than 30 pairs of polymorphic SSR primers in the amplification of *C. chinensis* collections and finally 20 microsatellite loci with excellent amplification among the collections were applied to the analysis of genetic diversity. We tested the null alleles with MICROCHECKER and found the frequency of null alleles is very low among these 20 microsatellite loci. Hence, null alleles may have merely slight effects on the results of genetic analysis in present study. The 20 microsatellite loci showed 149 alleles in 345 *C. chinensis* individuals and averaged 7.45 alleles per locus, and Shannon's information index averaged 1.015, and the values of *F*_*IS*_ and *F*_*ST*_ ranged from −0.456 to 0.791 and 0.110–0.472, with an average of 0.119 and 0.244, respectively, indicating that these SSR markers were informative and effective in differentiating among *C. chinensis* collections from different regions. An analysis of allelic diversity at the 20 microsatellite loci provided an insight into the population structure of *C. chinensis* from different regions (sampled in 2012 and 2013) representing important regional locations for legume production and storage in China. The present study is the first attempt at assessing the genetic diversity of *C. chinensis* using the SSR marker technique. It has demonstrated the validity and suitability of using SSR markers to detect genetic variation among *C. chinensis* populations.

Gene heterozygosity is an optimal parameter to measure genetic variation within populations. The average heterozygosity approximately reflects the level of genetic variation. The observed heterozygosity did not significantly deviate from expected heterozygosity at most of the loci, which confirmed validity of these loci among the collections in this study. The average expected and observed heterozygosity values for the 20 microsatellite loci and 23 *C. chinensis* populations were 0.4901 and 0.3389, respectively, suggesting a moderate level of genetic diversity among the samples. From this, we demonstrated that the *C. chinensis* population in ES (Hubei) had the highest genetic diversity, whereas the RD (Jiangsu) population had the lowest genetic diversity. The fixation index (*F*_*ST*_) reflects the degree of genetic differentiation among populations. *F*_*ST*_ is close to 0 when the genetic variation shows no sign of fixation between populations. However, *F*_*ST*_ is close to 1 when genetic differentiation is at a high level. Gene flow (*N*_*m*_) is considered one of the major factors in the homogenization of population genetic structure. Because gene flow and genetic drift are mutually antagonistic, exchange of genes can increase the amount of genetic variation within populations while decreasing differentiation between populations (Whitlock & McCauley, 1999). In this study, the *F*_*ST*_ values between populations ranged from 0.049 to 0.441, with corresponding *Nm* values varying from 9.788 to 0.633, indicating large differences in gene flow between *C. chinensis* collections from different regions. It is easy to understand why the maximum genetic differentiation appeared between the TS and RD populations, followed by the TS and CD populations, because there is typical geographical isolation between TS (Hebei) and RD (Jiangsu), and TS and Changde (Hunan), with distances of more than 1300 km from TS to RD and Changde. There are major differences in growth seasons, weather conditions, agricultural practices and legume varieties between these regions. In addition, based on our surveys, the Jilü series varieties and landraces were cultivated in TS, while Sulü and Tongcan series varieties and local breeds were mainly cultivated in RD. Moreover, mung bean and adzuki bean were main legumes in TS, whereas faba bean, pea and cowpea were cultivated widely in RD. Therefore, legume varieties varied greatly between the regions. In addition, the lack of legume seed exchange indeed existed between the regions due to different kinds of legumes, lack of communication and so on. In contrast, although the two areas are approximately 3000 km apart, the UM, Xinjiang and LC, Shanxi collections had the lowest genetic variation. This is understandable because frequent seed exchanges have occurred and the same varieties have been planted and stored in both areas for several years, furthermore, *C. chinensis* is apt to communicate and diffuse along with legume seeds by means of egg, larva, pupa or adult, which results in interaction of *C. chinensis* between the two areas and identical host selection pressures in both populations.

Based on AMOVA analysis, 23.3% of the total variation was found among regions, whereas 76.7% variance was attributable to population divergence within the regions. China is a large country with complex terrain and hence different levels of geographical isolation arise between *C. chinensis* populations from different regions, which enhances genetic variation among populations but decreases genetic differentiation within populations. However, frequent exchanges of legume seeds have taken place via trading and communication among some production areas for many years, which very likely includes *C. chinensis* eggs, nymphs, pupas, or adults. Therefore, the spread and diffusion of this pest increases the mating and gene flow among different collections and enhances heterogenization within populations.

As shown in the UPGMA clustering plot based on genetic distance between populations, the maximum genetic similarity was between the HF and RD collections, followed by the UM and LC, and ES and XY populations, whereas the WL and GA populations shared the minimum genetic similarity, followed by the WL and BB populations. The geographic distance between WL and GA is less than 300 km, but both of these regions are mountainous, and the food legume varieties planted in the two areas differ greatly; hence, the host selection pressures differ and there is limited communication, which has resulted in geographical isolation and limited exchange of genetic information between the two populations.

The pattern of three groups from PCA analysis looked different from cluster result with four genetic groups. Compared with figs 3 and 4, Cluster I and Cluster II in fig. 3 were combined to the first principal component in fig. 4, whereas the Cluster III and Cluster IV coincided with the other two components, respectively. However, we think the results from these two methods are similar and comparable. It is not hard to see that 23 populations can also be classified into three genetic groups at a similarity coefficient of 0.135. In the present study, UPGMA cluster and PCA analysis were conducted based on geographic populations (subgroups) and individuals, respectively. As discussed above, there often exists gene exchange among *C. chinensis* from these regions and then leads to a decline in genetic differentiation therefore individuals from Cluster I and Cluster II comprised a principal component.

High adaptability to different environments or hosts is usually correlated with abundant genetic diversity. However, *C. chinensis* is apt to diffuse and spread by way of seed trade and communication, which results in frequent gene flow and decreases heterogeneity among collections from different regions in China. Therefore, our findings that there is high adaptability and moderate genetic diversity among *C. chinensis* populations are understandable and reasonable, and should help to develop a control strategy for *C. chinensis*.
